# Cracks in the Foundation: The Association of Physical Condition of School Facilities With Absenteeism and Test Scores in Maryland

**DOI:** 10.1111/josh.70131

**Published:** 2026-02-26

**Authors:** Catherine H. Gong, Richard Lofton, Priyanka Fernandes, Odis Johnson, Joshua M. Sharfstein

**Affiliations:** ^1^ Johns Hopkins Bloomberg School of Public Health Baltimore Maryland USA; ^2^ Johns Hopkins University School of Education Baltimore Maryland USA; ^3^ Johns Hopkins University School of Medicine Baltimore Maryland USA

**Keywords:** absenteeism, school infrastructure, test scores

## Abstract

**Background:**

Poor physical conditions of school facilities are linked to poor health, lower test scores, and higher rates of chronic absenteeism.

**Methods:**

We conducted a cross‐sectional analysis using data on physical conditions of school facilities, absenteeism, and test scores for 1266 K‐12 school facilities representing 1388 schools (which may share school facilities) in Maryland. We analyzed the results by race and ethnicity of students and by the area deprivation index.

**Results:**

Students in schools in the 10th decile for poor physical conditions experienced significantly higher rates of chronic absenteeism and significantly lower SAT, ACT, and Maryland state test scores. Black and Hispanic students were significantly more likely to attend these schools. The significant association between poor school facility conditions and educational outcomes is limited to communities with high socioeconomic deprivation.

**Implications for School Health Policy, Practice, and Equity:**

Policymakers should consider investments in school infrastructure in under‐resourced communities to close educational gaps and help every child succeed.

**Conclusions:**

In Maryland communities with high socioeconomic deprivation, poor school facility conditions are associated with greater absenteeism and lower test scores, disproportionately affecting Black and Hispanic children.

## 1 Background

The physical infrastructure of schools has a major impact on both education and health. Many U.S. public school facilities suffer from poor conditions such as leaking roofs, inadequate temperature regulation, improper ventilation, and poor water filtration [1], which are hypothesized to impact student outcomes through a range of mechanisms that are independent of other school dimensions, such as staffing and instructional quality [2]. Directly, poor conditions of school facilities can cause or exacerbate student illness that interferes with learning, including asthma and other respiratory conditions [3, 4, 5, 6]. Indirectly, such conditions undermine the learning environment through facility closures [7], less teacher satisfaction and retention [8, 9], and worse school climate [10, 11]. Poor conditions of school facilities have been consistently linked to decreased school attendance and absenteeism [12, 13, 14], which are associated with both lower academic achievement and worse long‐term health outcomes [15, 16]. The importance of school infrastructure for educational success has also been recognized in low‐ and middle‐income countries [17, 18, 19].

The consequences of inadequate school infrastructure are not spread evenly across the U.S. population. School facilities located in disadvantaged areas are more likely to be older and in worse condition than those in wealthier districts [20, 21]. The impacts are also distributed unequally across race and ethnicity. Black and Hispanic students are more likely to attend schools with higher levels of need and less funding than White students [22, 23], reflecting the long‐term, concentrated debt experienced by minoritized communities [24, 25, 26, 27].

These issues have yet to be studied at a statewide level. To assess the impacts of school infrastructure in a large population of public school students, we conducted the first statewide study of school facility conditions and educational outcomes. In 2020 and 2021, the Maryland Interagency Commission on School Construction (IAC) surveyed the conditions of all K‐12 public school facilities within the state as part of the Maryland Statewide Facilities Assessment [28]. Using the data from this survey, we assessed the relationship between school facility conditions and educational outcomes, with attention to the race and ethnicity of students and socioeconomic status of communities.

## 2 Methodology

In this study, the term school facility refers to a building surveyed as part of the Maryland Statewide Facilities Assessment. The term school refers to an educational unit. More than one school (such as two specialized high schools) may be present in a single school facility. We conducted a cross‐sectional analysis of the conditions of school facilities with educational outcomes in Maryland in 1420 K‐12 Maryland schools. The analysis proceeded in three steps. First, we assessed whether the schools in facilities in the worst conditions were more likely to see higher rates of absenteeism and lower test scores than other Maryland schools. Second, we evaluated whether schools with facilities in poor conditions were more likely to have a significant percentage of Black and Hispanic students. Third, we examined whether the relationship between school facility conditions and educational outcomes persists after controlling for socioeconomic status of the surrounding community.

### 2.1 Instrumentation

#### 2.1.1 Measuring School Facility Conditions

To assess the physical conditions of school facilities, we created a measurement called the current Maryland Condition Index or cMDCI. This score includes all but one of the elements of the IAC's Maryland Condition Index.

To calculate the Maryland Condition Index, the IAC creates a weighted average of subscores for each school component (such as an HVAC system or roof). Each subscore is the product of (1) the replacement value of the component, as stipulated by the IAC; (2) the “percentage of the expected useful lifespan that is depleted,” as determined by inspectors; and (3) the factor that represents the potential to interfere with the school environment, as determined by inspectors [29]. These factors range from 3.5 for “critical issues that pose immediate threats to the life, health, or safety of persons within the facility” to 0.25 for “Systems that are within the expected life cycle and do not require replacement.” The IAC includes in the Maryland Condition Index an additional subscore for space that is assumed to be needed in the future based on projected enrollment growth.

To focus on the current conditions of the school facilities, we calculated a Maryland Condition Index without the additional space subscore, calling the resulting measure the cMDCI.

#### 2.1.2 Measuring Absenteeism

Our measure of absenteeism was the percentage of students from elementary through high school who were chronically absent from each school during the 2021–2022 academic year. This information was sourced from the Maryland State Department of Education, which defines a chronically absent student as one who misses ≥ 10% of school days.

#### 2.1.3 Measuring Test Scores

For test scores, we separated high schools from elementary and middle schools and again sourced our data from the Maryland State Department of Education. For each high school, we used the mean ACT and SAT scores during the 2021–2022 academic year. For each elementary and middle school, we used the percentage of students who tested as proficient under the Maryland Comprehensive Assessment Program (MCAP) for English Language Arts (ELA) and Math separately during the 2021–2022 academic year.

To calculate the percentage of students who were proficient in the MCAP ELA, we divided the total number of students per school who scored as proficient by the number of students who took the exam per school. Likewise, to calculate the percentage of students who were MCAP Math proficient, we divided the number of students per school who scored as proficient for their grade level by the number of students who took the exam per school. Due to variation in the number of students taking these classes, we excluded middle school Algebra 1, Algebra 2, and Geometry proficiency results from this calculation. We did not include high school MCAP results because ELA proficiency was only available for grade 10, while some students take this test in middle school and others in high school.

### 2.2 Procedure and Data Analysis

#### 2.2.1 Association Between School Facility Conditions, Absenteeism, and Test Scores

We hypothesized that associations with absenteeism and test scores would not be linear and would instead be observed at a certain threshold of school facility conditions in Maryland. To assess whether such a threshold exists, we divided our sample of Maryland public school facilities into deciles by cMDCI score. The first decile included the school facilities in the best condition, while the 10th decile included the school facilities in the worst condition. We created deciles in school facility conditions for each analysis: (1) all school facilities and absenteeism; (2) high schools and ACT/SAT; and (3) elementary/middle schools and MCAP scores.

For each decile, we calculated the mean percentage of students who were chronically absent, weighted by the number of students enrolled in each school. 1266 Maryland school facilities were analyzed out of a total of 1378 school facilities (112 school facilities were excluded due to incomplete data, reflecting that these facilities may be supplementary spaces and not schools). For each decile of high schools that were grouped by school facility condition score, we calculated the mean ACT and SAT scores, weighted by the number of students who took the test in each decile. After 42 high schools were excluded due to missing ACT and SAT data, 177 high schools in 177 school facilities were analyzed. For each decile of elementary and middle schools grouped by school facility condition score, we calculated the mean percentages of elementary and middle school students who were MCAP ELA proficient and MCAP Math proficient, weighted by the number of students who took the test. 1098 elementary and middle schools in 1031 school facilities were analyzed, with 103 schools excluded due to missing MCAP data. We then compared the 6th, 7th, 8th, 9th, and 10th deciles with the average outcomes of the preceding deciles to determine if there is a difference in absenteeism or test scores. For this comparison, we used Welch's *t*‐test, with significance identified at *p* < 0.05. We used R version 4.2.2 for the analysis.

#### 2.2.2 Demographic Assessment

After establishing a threshold effect in the association between school facility conditions, absenteeism, and test scores, we evaluated whether Black students, Hispanic students and White students were more likely to be in the school facilities with conditions associated with greater absenteeism, and lower test scores. For this analysis, we assessed enrollment by race in our sample of all Maryland public schools, using chi‐square tests in R version 4.2.2.

#### 2.2.3 Relationship With Area Disadvantage

We analyzed how socioeconomic disadvantage may relate to the relationship between school facility conditions and education.

For the measurement of area disadvantage, we used the 2021 area deprivation index value associated with the Census block group in which each school was located. The area deprivation index is based on a measure created by the Health Resources and Services Administration and includes factors for the theoretical domains of income, education, employment, and housing quality [30, 31]. Then, we created a binary variable to represent whether or not a school was in Deciles 6–10 of the Area Deprivation Index, reflecting whether or not a school was located in one of the more disadvantaged neighborhoods.

We similarly classified schools as whether or not they were in Decile 10 of cMDCI score, absenteeism, SAT score, MCAP ELA score, or MCAP Math score. Mean ACT scores were not assessed in this step due to inadequate variation. Using the constructed binary variables, we first assessed the relationship between school facility conditions and educational outcomes of schools above versus below median area disadvantage using chi‐square tests in R version 4.2.2.

## 3 Results

### 3.1 Absenteeism

**FIGURE 1:**
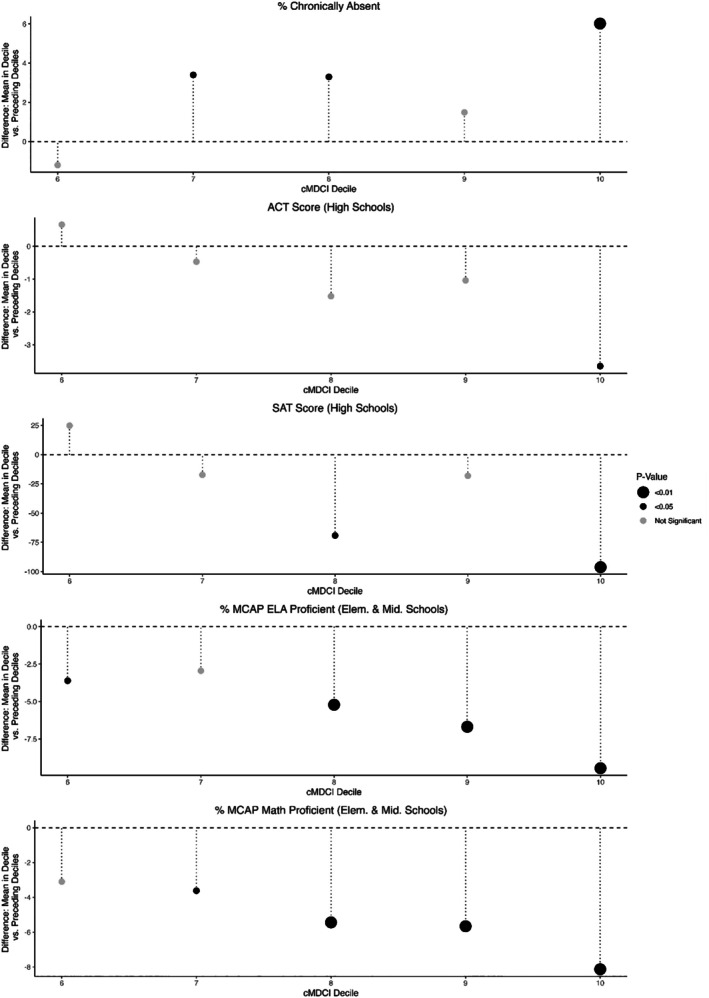
Mean difference in educational outcomes between decile and preceding deciles of school condition score. Data available in Table S1.

Each decile of school facility conditions by cMDCI score was comprised of 125 or 126 school facilities with between 66,003 and 97,414 students (Table 1). Worse school facility conditions were associated with greater absenteeism. The mean percentage of students in 10th decile school facilities who were chronically absent was 6.0% higher than the mean of the other 90% of school facilities (36.3% vs. 30.2% respectively, *p* < 0.001). Similar associations were found for schools in the 7th and 8th deciles (Figure 1).

**TABLE 1:** Number of school facilities, schools, and students analyzed in absenteeism, ACT/SAT, and MCAP analyses.

	Absenteeism analysis			ACT/SAT analysis				MCAP analysis			
cMDCI decile	School facilities	Schools	Students	High school facilities	High schools	Students ACT	Students SAT	Elem/middle school facilities	Elem/middle schools	Students MCAP ELA	Students MCAP math
1	126	140	92,720	18	18	196	2481	97	110	34,851	31,807
2	126	136	97,414	18	18	340	3675	105	110	40,247	37,175
3	126	131	90,370	17	17	392	3058	108	110	40,012	35,958
4	125	130	84,913	18	18	397	3990	104	109	35,966	32,289
5	126	138	96,576	18	18	410	3320	104	110	39,560	35,021
6	126	133	81,604	17	17	538	3932	107	110	38,485	35,209
7	125	135	77,107	18	18	134	2844	106	109	36,190	33,310
8	126	145	70,849	17	17	108	2392	104	110	34,446	32,198
9	126	142	66,003	18	18	166	2077	102	110	28,201	27,233
10	126	148	69,372	18	18	112	2634	94	110	28,605	26,317

*Note:* The deciles of school conditions were calculated separately for the absenteeism (based on school facilities), ACT/SAT (based on high schools), and MCAP analyses (based on elementary/middle schools). For eight schools with more than one building, a weighted average by square footage for the facility score was used for the school building condition. More than one school may be located in a school facility. There are fewer students in the test score analyses than in the absenteeism analysis because not all students take the standardized tests.

### 3.2 ACT/SAT Scores

Each decile of school facility conditions by cMDCI score included 17 or 18 high schools and represented between 108 and 538 ACT takers and between 2077 and 3990 SAT takers (Table 1). Worse school facility conditions were associated with lower SAT and ACT scores. The mean SAT score of 10th decile high schools was significantly lower than the mean SAT score of all other high schools (992.9 vs. 1089.2, respectively, *p* < 0.001). Similar associations were found for schools in the 8th decile. The mean ACT score of 10th decile high schools was 3.6 points lower than the mean ACT score of all other high schools (21.4 vs. 25.1, respectively, *p* = 0.04) (Figure 1).

### 3.3 MCAP Proficiency

Each decile of school facility conditions by cMDCI score included 109 or 110 elementary and middle school facilities and between 28,201 and 40,247 MCAP test takers in English Language Arts (ELA) and between 26,317 and 37,175 MCAP test takers in math (Table 1). The mean percentage of students in the 10th decile of elementary and middle schools who were MCAP proficient (ELA 35.9%, Math 16.2%) was significantly lower (ELA *p* < 0.001, Math *p* < 0.001) than the mean of the other 90% of elementary and middle schools (ELA 45.4%, Math 24.2%) (Figure 1).

### 3.4 Demographic Assessment

Black and Hispanic students are significantly more likely to attend schools associated with greater absenteeism and lower test scores—specifically, those in the 10th decile of poor school facility conditions by cMDCI score. Specifically, 11.1% of Black students in Maryland, compared to 7.1% of non‐Black students, attended a school in the 10th decile. Black students were 1.57 times more likely to be enrolled in a 10th decile school than their non‐Black counterparts (*p* < 0.001). Hispanic students in Maryland were also more likely to attend a school in the 10th decile for school facility conditions than their non‐Hispanic counterparts (8.8% vs. 8.2% respectively, *p* < 0.001).

In contrast, 6.6% of White students in Maryland, compared to 9.3% of non‐White students, attended a school with the worst conditions. White students were 30% less likely to be enrolled in a 10th decile school than their non‐White counterparts (*p* < 0.001).

### 3.5 Socioeconomic Disadvantage

Table 2 displays the odds of a school having an education outcome in the 10th decile given that its building condition is in the 10th decile, grouped by whether the school area is less disadvantaged (Decile 1–5) or more disadvantaged (Decile 6–10). Across all four education outcomes assessed, among schools that are in more disadvantaged areas, school facility conditions were associated with educational outcomes. For example, a school in a more disadvantaged area in the 10th decile of building condition had 2.05 greater odds of also having chronic absenteeism in the 10th decile (*p* = 0.009).

**TABLE 2:** Relationship between poor school conditions and educational outcomes, by social disadvantage.

	Low social disadvantage schools	High social disadvantage schools
Odds of highest chronic absenteeism in poorest condition schools	0	2.05**
Odds of lowest SAT scores in poorest condition schools	0	1.91
Odds of lowest MCAP ELA proficiency in poorest condition schools	3.99	2.34**
Odds of lowest MCAP Math proficiency in poorest condition schools	0	2.50**

*Note:* “Highest” and “lowest” refer to decile 10 of the education outcome. Low social disadvantage schools are located in areas considered to have an area deprivation index below the median. High social disadvantage schools are located in areas considered to have an area deprivation index above the median. Data available in Table S2.

*p < 0.05; **p < 0.01; ***p < 0.001.

## 4 Discussion

This analysis finds that attending Maryland schools with the worst physical conditions is associated with higher rates of absenteeism, lower ACT and SAT scores, and lower MCAP proficiency. The absolute value of these differences is material, but not overwhelming: 6 percentage points on absenteeism, 3.5 points on the ACT, 100 points on the SAT score, and 8%–10% difference in English and math proficiency. Furthermore, the association is limited to areas with greater socioeconomic deprivation, suggesting that wealth and other advantages may be able to mitigate the impact of adverse school facility conditions on school performance. Practically, a school facility at risk might be located next to boarded‐up homes, with multiple systems (such as roof and HVAC) that are years past their expected duration, and hazards in the playground or athletic fields.

While this study cannot demonstrate causality, there is evidence that adverse school facility conditions are impairing educational progress in poorer areas of Maryland. The first is the direct impact of these conditions on time in school, as adverse heating and cooling conditions have led to school closures during inclement weather. For example, over a recent 5‐year period, Baltimore City public school students missed a total of 221,000 days of school because of facilities issues [7]. The second is interference with the school environment. Students themselves have expressed concerns with failed temperature regulation, unsanitary bathrooms, and pests, and have noted that adverse conditions impair their ability to concentrate during testing such as the SAT [32].

The third is the demoralization of students. In interviews, students have stated that they associate the conditions of their schools with the value that society places on their learning, undermining their motivation to succeed. As one student stated, “So I believe it's because of our skin color and because they don't really care about us because if they did, then they would actually fix our buildings.” [32] This third factor may particularly affect young people in poorer areas and help explain why schools in areas of less socioeconomic deprivation may be less susceptible to the impact of adverse conditions.

In Maryland, Black and Hispanic students are disproportionately more likely to attend schools where the conditions are so poor as to be associated with absenteeism and lower test scores. These findings must be understood in the context of fundamental inequities in access to educational resources, which in part reflect many years of segregation and other historical injustices [33, 34]. Poor physical conditions of schools can be seen as another element of systemic discrimination affecting minoritized communities, contributing to the ongoing racial education gaps [35].

### 4.1 Implications for School Health Policy, Practice, and Equity

Policymakers interested in closing educational achievement gaps often focus on educational strategies and annual budgets. Our results suggest that attention must also be paid to the major investments needed to upgrade physical infrastructure. Indeed, evidence from Los Angeles suggests major investments in school capital funding are linked to advances in educational outcomes [36].

The relationship between poor school facility conditions and lower test scores and greater absenteeism is evident in the more disadvantaged communities represented in our data. These findings highlight the importance of adequate spending on school infrastructure in these areas, which will likely require funding streams besides local tax revenues. The diversity of state and local arrangements for school funding and governance means that the specific implications for policy may differ across the country.

### 4.2 Limitations

This analysis has several limitations. First, we focused on the 2021–2022 school year amid the continuing disruption of the COVID‐19 pandemic, which was associated with increased rates of absenteeism and could have affected some of the results on achievement [37]. In Maryland, as nationally, these challenges have persisted, suggesting that pandemic era analyses may have greater relevance to the current situation than pre‐pandemic studies. Second, we were unable to include additional school‐level potential confounders in the analysis that affect educational outcomes, such as teacher quality and school funding levels. As a result, we were not able to determine the relationship between school facility conditions, educational outcomes, and these other factors. Third, the analysis did not distinguish between magnet and non‐magnet schools, which may distort results on test scores and absenteeism. However, some facilities for magnet schools in Maryland are in particularly poor condition, suggesting that the results here may understate the association between school conditions and achievement.

## 5 Conclusion

Children in Maryland school facilities with the worst physical conditions are less likely to excel academically. Addressing the physical conditions of school facilities, particularly in areas of socioeconomic deprivation, may close achievement gaps and facilitate educational success.

## Ethics Statement

Preparation of this paper did not involve primary research or data collection involving human subjects, and therefore, no institutional review board examination or approval was required.

## Conflicts of Interest

The authors declare no conflicts of interest.

## Supporting information


**Table S1:** Mean educational outcomes by cMDCI school decile using Welch's *t*‐test for differences between preceding deciles.
**Table S2:** Odds of education outcome in the 10th decile given school building condition in the 10th decile, above/below area deprivation index (ADI) median.

## Data Availability

The data that support the findings of this study are available from the corresponding author upon reasonable request.
