# Supplementary material for: Cracks in the Foundation: The Association of Physical Condition of School Facilities With Absenteeism and Test Scores in Maryland
Source: J Sch Health. 2026 Feb 26;96(4):e70131. doi: 10.1111/josh.70131 (PMC12945478; doi:10.1111/josh.70131)
Supplement: Supplementary file 1 — Table S1: Mean educational outcomes by cMDCI school decile using Welch's t‐test for differences between preceding deciles. Table S2: Odds of education outcome in the 10th decile given school building condition in the 10th decile, above/below area deprivation index (ADI) median. [file JOSH-96-0-s001.docx]

**Supplementary File**

Table 1S: Mean educational outcomes by cMDCI school decile using Welch’s t-test for differences between preceding deciles.

| **cMDCI Decile** | **Mean in Decile** | **Mean Preceding Deciles** | **P-Value** |
| --- | --- | --- | --- |
| **% Chronically Absent** | | | |
| 6 | 28.37 | 29.57 | 0.322 |
| 7 | 32.79 | 29.39 | 0.028 |
| 8 | 33.11 | 29.81 | 0.020 |
| 9 | 31.64 | 30.15 | 0.202 |
| 10 | 36.30 | 30.28 | **<0.001** |
| **ACT Score (High Schools)** | | | |
| 6 | 25.79 | 25.13 | 0.602 |
| 7 | 24.81 | 25.28 | 0.552 |
| 8 | 23.74 | 25.26 | 0.083 |
| 9 | 24.15 | 25.19 | 0.591 |
| 10 | 21.48 | 25.13 | 0.042 |
| **SAT Score (High Schools)** | | | |
| 6 | 1119.16 | 1094.34 | 0.536 |
| 7 | 1081.84 | 1099.11 | 0.497 |
| 8 | 1027.85 | 1097.00 | 0.031 |
| 9 | 1072.47 | 1090.56 | 0.593 |
| 10 | 992.95 | 1089.21 | **<0.001** |
| **% MCAP ELA Proficient (Elem. & Mid. Schools)** | | | |
| 6 | 44.01 | 47.62 | 0.042 |
| 7 | 44.07 | 47.02 | 0.102 |
| 8 | 41.40 | 46.62 | **0.004** |
| 9 | 39.33 | 46.02 | **<0.001** |
| 10 | 35.98 | 45.44 | **<0.001** |
| **% MCAP Math Proficient (Elem. & Mid. Schools)** | | | |
| 6 | 23.33 | 26.43 | 0.087 |
| 7 | 22.28 | 25.90 | 0.041 |
| 8 | 19.96 | 25.40 | **0.003** |
| 9 | 19.10 | 24.76 | **0.001** |
| 10 | 16.12 | 24.25 | **<0.001** |

Note: In P-Value column, black indicates p < 0.05. **Black bold** indicates p < 0.01.

Table 2S: Odds of education outcome in the 10th decile given school building condition in the 10th decile, above/below Area Deprivation Index (ADI) median.

|  |  | | **% Chronically Absent Decile 10** | | Odds Ratio |
| --- | --- | --- | --- | --- | --- |
|  |  |  | *Yes* | *No* |  |
| ADI Decile 1-5 | cMDCI Decile 10 | *Yes* | 0 | 11 | 0 |
|  |  | *No* | 40 | 614 |  |
| ADI Decile 6-10 | cMDCI Decile 10 | *Yes* | 24 | 83 | 2.05 ** |
|  |  | *No* | 58 | 412 |  |
|  |  | | **Mean SAT Score Decile 10** | | Odds Ratio |
|  |  |  | *Yes* | *No* |  |
| ADI Decile 1-5 | cMDCI Decile 10 | *Yes* | 0 | 7 | 0 |
|  |  | *No* | 5 | 81 |  |
| ADI Decile 6-10 | cMDCI Decile 10 | *Yes* | 3 | 9 | 1.91 |
|  |  | *No* | 10 | 58 |  |
|  | | | **% MCAP ELA Proficient Decile 10** | | Odds Ratio |
|  |  |  | *Yes* | *No* |  |
| ADI Decile 1-5 | cMDCI Decile 10 | *Yes* | 2 | 9 | 3.99 |
|  |  | *No* | 29 | 523 |  |
| ADI Decile 6-10 | cMDCI Decile 10 | *Yes* | 24 | 74 | 2.34 ** |
|  |  | *No* | 52 | 376 |  |
|  | | | **% MCAP Math Proficient Decile 10** | | Odds Ratio |
|  |  |  | *Yes* | *No* |  |
| ADI Decile 1-5 | cMDCI Decile 10 | *Yes* | 0 | 14 | 0 |
|  |  | *No* | 31 | 518 |  |
| ADI Decile 6-10 | cMDCI Decile 10 | *Yes* | 24 | 70 | 2.50 ** |
|  |  | *No* | 52 | 380 |  |

*p<0.05 **p<0.01 ***p<0.001
